# Siphonaptera of Canada

**DOI:** 10.3897/zookeys.819.25458

**Published:** 2019-01-24

**Authors:** Terry D. Galloway

**Affiliations:** 1 Department of Entomology, University of Manitoba, Winnipeg, Manitoba, R3T 2N2, Canada University of Manitoba Winnipeg Canada

**Keywords:** biodiversity assessment, Biota of Canada, fleas, Siphonaptera

## Abstract

There are currently 154 species of fleas recorded in Canada in four superfamilies and seven families. Only two species have been added to the list since the previous summary by Holland (1979) one of which is unlikely to be established in Canada. There have been a number of significant nomenclatural changes since then most notable of which is the split of the Hystrichopsyllidae into two families Hystrichopsyllidae and Ctenophthalmidae. An additional 23 species may eventually be recorded based on presence of suitable hosts and proximity to known distributions. Six species are introduced and one species is adventive. Although total diversity is reasonably well known there are numerous gaps in distribution of fleas throughout the country. Barcode Index Numbers are available for only 22 species of fleas collected in Canada.

Fleas are a relatively small group, with more than 2200 species known worldwide. Species in Canada range in size from <2 mm (the introduced sticktight flea, *Echidnophagagallinacea* (Westwood)) to >9 mm (*Hystrichopsyllaschefferi* Chapin, likely the largest flea in the world). Fleas have profound medical and veterinary significance, and are best known for their role as vectors for the bacterial agent of bubonic plague. Their consequent historic impact on humans and society has been immeasurable. Flea vectors and bubonic plague have been recorded in Canada, and although known to occur in humans, have not been of major concern in recent years. However, the fear of plague epidemics was the stimulus for some of the early work on fleas in western Canada. As a result, our knowledge of the flea fauna of Canada is very good, largely through the efforts of George P. Holland. He produced two monographs (Holland 1949, 1985), the most recent of which included nearly all species recorded for Canada, as well as those for Alaska and Greenland. Today, fleas are mostly regarded as a source of annoyance to humans and to their pets. For example, the introduced cat flea is widespread, and a primary target for pest control in the home.

The following account is for the 154 species known to occur in Canada (Table 1). There have been some nomenclatural changes since Holland (1979, 1985), but only two species, *E.gallinacea* and *Myodopsyllaborealis* Lewis, have been added to our fauna since that time (Chilton et al. 2000, Galloway et al. 2000). With these exceptions in mind, it is possible to identify the majority of taxa in Canada using Holland’s more recent monograph (Holland 1985). The number of species recorded in Canada represents about 51% of the 303 species found in North America (Lewis and Eckerlin, in press). By far the greatest diversity of species occurs in British Columbia, and more than two thirds of species in Canada are found only in the provinces west of Ontario. There are six species of fleas introduced into North America north of Mexico that have been recorded in Canada (Pulicidae, 3; Leptopsyllidae, 1; Ceratophyllidae, 2), mostly ectoparasites of synanthropic rodents, poultry, and pets. One species of Pulicidae, *E.gallinacea*, is adventive on migratory birds from the United States and unlikely to become established in Canada.

The higher classification of Lewis (1993a, 1998, 2000) is adopted here. Because of his recognition of the Ctenophthalmidae at the family level, including the Stenoponiinae, Neopsyllinae, Rhadinopsyllinae, Ctenophthalminae, Doratopsyllinae, and Anomiopsyllinae, compared to Holland’s Ctenophthalminae (as part of the Hystrichopsyllidae), the tables here and in Holland (1979) do not compare readily. As well, Holland (1979) cited all taxa, including subspecies, known for Canada as well as for Alaska and Greenland, further complicating comparison of numbers of taxa from 1979 to now. Taxonomic and nomenclatural changes introduced by Smit (1983) in his treatment of the Ceratophyllidae were published too late for Holland to consider and make changes to his 1985 monograph, though these changes were mostly at the generic level and have no effect on numbers of taxa in Holland (1985). There are recent monographs and regional lists of fleas in biogeographic areas outside Canada, where flea species not yet recorded for Canada occur. These include Traub et al. (1983), Lewis et al. (1988), Haas et al. (1989), Lewis (1990), Larson (1997), and Lewis (1998). The papers of RE Lewis (1972, 1973, 1974, 1975, 1993b, Lewis and Lewis 1985) on distribution and host preferences in fleas and on classification of the Siphonaptera are valuable references. As well, Lewis and colleagues published several recent reviews of genera of fleas in North America (Lewis 2000, 2002, 2003, 2008a, b, 2009, Lewis and Galloway 2001, Lewis and Haas 2001, Lewis and Jameson 2002, Lewis and Wilson 2006) wherein there are relevant nomenclatural changes from taxa cited by Holland (1985). In addition, volumes I-V of the Illustrated Catalogue of the Rothschild Collection of Fleas (Hopkins and Rothschild 1953–1971) are essential companions for anyone studying fleas in Canada.

The flea fauna of Canada is relatively well known; it is expected that at least another 23 species may be discovered, more than half of these in the family Ceratophyllidae. These estimates were generated by considering the fauna of adjacent jurisdictions (northern United States, including Alaska) that include species with ranges in close proximity and for which known hosts occur in Canada. I know of no undescribed species of fleas in Canada.

There are enormous gaps in the known ranges of various species across the country. For example, *Tarsopsyllaoctodecimdentatuscoloradensis* (Baker) is a nest flea of red squirrel, *Tamiasciurushudsonicus* (Erxleben), and *Kueichenlipsyllaatrox* (Jordan) is a winter-active flea that infests mustelids. Known records are few and widely scattered. They no doubt occur in many other parts of Canada, but apparent rarity and gaps in distribution are probably the result of insufficient targeted collecting effort. There is also scant information on the seasonal dynamics and life histories of fleas, though there are some recent studies on important aspects of ecology and flea/host interactions (e.g., Reichardt and Galloway 1994, Lindsay and Galloway 1997, 1998, Thomas and Shutler 2001, Perez-Orella and Schulte-Hostedde 2005, Shutler and Campbell 2007, Gorrell and Schulte-Hostedde 2008, Guderham and Schulte-Hostedde 2011, Waterman et al. 2014, Raveh et al. 2015). Larval taxonomy is in its infancy in Canada (but see Elbel 1991, Pilgrim and Galloway 2000, 2007, Galloway and Pilgrim 2001, Harriman et al. 2011). There are extensive or partial larval descriptions for only 16 of the taxa found in Canada, seven of which are for introduced and adventive species.

The Canadian fauna has been poorly characterised using DNA barcodes; there are only about 14% as many BINs as there are described species in Canada, most of which (18 of 22) are from Ceratophyllidae (Table 1). Recent and current studies are focussed on a small number of flea taxa, and there are few broad surveys being undertaken where additional taxa for DNA barcoding could be collected. This represents a significant gap in our knowledge of the fleas of Canada. There are certainly taxonomic problems that could benefit from careful research in which DNA barcoding is a component. For example, there are four species of *Ceratophyllus* that infest cliff swallows (*Petrochelidonpyrrhonota* Veillot) in Canada: *Ceratophylluscelsuscelsus* Jordan, *C.scopulorum* Holland, *C.calderwoodi* Holland, *C.arcuegens* Holland. Although their geographic distributions are largely allopatric, there are critical areas of overlap (Galloway 1988). DNA barcoding might be a valuable tool to examine species status among these taxa. It is important that people continue to collect adult fleas and larvae from identified hosts and their nests throughout the country to add to our understanding of this important group of ectoparasites.

**Table 1.** Census of Siphonaptera in Canada.

Taxon^1^	Adjusted no. species reported in Holland (1979)^2^	No. species currently known from Canada^3^	No. BINs^4^available for Canadian species	Est. no. undescribed or unrecorded species in Canada	General distribution by ecozone^5^ and host range	Information sources
**Superfamily Pulicoidea**						
Pulicidae	7^6^	8 (3)	1	2	potentially all ecozones; wide variety of mammals and birds	Holland 1949, 1985, Galloway et al. 2000, 2002; WRME^7^
**Superfamily Vermipsylloidea**						
Vermipsyllidae	3	3	0	2	Western Interior Basin, Boreal Plains, Mixedwood Plains, Pacific Maritime, Montane Cordillera, Prairies, Atlantic Maritime; mostly carnivores	Holland 1949, 1985; WRME
**Superfamily Hystrichopsylloidea**						
Hystrichopsyllidae ^8^	6	6	0	0	all ecozones south of taiga; insectivores, small rodents	Holland 1949, 1985; WRME
Ctenophthalmidae ^8^	50	51^9^	3	3	all except Arctic ecozones; insectivores, rodents	Holland 1949, 1985, Lewis and Haas 2001, Morrone and Acosta 2006; WRME
**Superfamily Ceratophylloidea**						
Ischnopsyllidae	4	5	0	0	transcontinental exclusive of Arctic and alpine areas; specific to bats	Holland 1949, 1985, Chilton et al. 2000; WRME
Leptopsyllidae	14	14 (1)	0	3	all except Arctic; rodents, lagomorphs, birds	Holland 1949, 1985; WRME
Ceratophyllidae	67	67 (2)	18	13	all ecozones; mainly small mammals and birds	Holland 1949, 1985, Lewis 1990, 1993a, b, 2000, 2002, 2003, 2007, 2008a, b, 2009, Lewis and Galloway 2001, Lewis and Haas 2001, Lewis and Jameson 2002, Lewis and Wilson 2006, Lewis and Eckerlin 2013; WRME
**Total**	**151**	**154 (6)**	**22**	**23**		

^1^Classification follows Lewis (2000). ^2^Holland (1979) included subspecies in the numbers he reported, many of which have since been suppressed. Holland also included species recorded in Alaska and Greenland, but not in Canada. Numbers of species in this column have been adjusted to include only species Holland (1985) recorded as occurring in Canada. ^3^Numbers in parentheses represent the numbers of introduced species included in the total for each family, exclusive of the non-native, *E.gallinacea*, which may disperse into Canada on birds from the USA. ^4^Barcode Index Number, as defined in Ratnasingham and Hebert (2013). ^5^See figure 1 in Langor (2019) for a map of ecozones. ^6^Holland (1979) reported eight species of Pulicidae, but it appears he included *Actenopsyllasuavis* Jordan & Rothschild in that total; he reported no specimens that had actually been collected in Canada or Alaska. Only those seven species for which there are Canadian records are included here. ^7^J.B. Wallis/R.E. Roughley Museum of Entomology, Department of Entomology, University of Manitoba, Winnipeg, MB. ^8^Holland (1979) treated this group of fleas as one family, Hystrichopsyllidae, though it is currently separated into two families, Hystrichopsyllidae and Ctenophthalmidae. Numbers of species in each family in 1979 are based on those reported by Holland (1985). ^9^Totals adjusted to accommodate nomenclatural changes to the genera, *Catallagia* (Lewis and Haas 2001) and *Corrodopsylla* (Morrone and Acosta 2006).
